# GdVO_4_:Eu^3+^ and LaVO_4_:Eu^3+^ Nanoparticles Exacerbate Oxidative Stress in L929 Cells: Potential Implications for Cancer Therapy

**DOI:** 10.3390/ijms252111687

**Published:** 2024-10-30

**Authors:** Yuriy Kot, Vladimir Klochkov, Volodymyr Prokopiuk, Olha Sedyh, Liliya Tryfonyuk, Ganna Grygorova, Nina Karpenko, Oleksandr Tomchuk, Kateryna Kot, Anatolii Onishchenko, Svetlana Yefimova, Anton Tkachenko

**Affiliations:** 1 Department of Biochemistry, V.N. Karazin Kharkiv National, 4 Svobody Sq, 61022 Kharkiv, Ukraine; kot.juriy@gmail.com (Y.K.); kate.v.kot@gmail.com (K.K.); 2 Department of Nanostructured Materials, Institute for Scintillation Materials of the National Academy of Sciences of Ukraine, 60 Nauky Ave, 61072 Kharkiv, Ukraine; klochkov@isma.kharkov.ua (V.K.); sedykholga4424@gmail.com (O.S.); grigorova@isma.kharkov.ua (G.G.); nina.a.karpenko@gmail.com (N.K.); 3 Department of Cryobiochemistry, Institute for Problems of Cryobiology and Cryomedicine of the National Academy of Sciences of Ukraine, 23 Pereyaslavskaya Str., 61015 Kharkiv, Ukraine; v.yu.prokopiuk@gmail.com (V.P.); ai.onishchenko@knmu.edu.ua (A.O.); 4 Research Institute of Experimental and Clinical Medicine, Kharkiv National Medical University, 4 Nauky Ave, 61022 Kharkiv, Ukraine; 5 Institute of Health, National University of Water and Environmental Engineering, 11 Soborna Str., 33028 Rivne, Ukraine; liliya_tryfonyuk@yahoo.pl; 6 ISIS Neutron and Muon Source, Rutherford Appleton Laboratory, Harwell Oxford, Didcot OX11 0QX, UK; oleksandr.tomchuk@stfc.ac.uk; 7 BIOCEV, First Faculty of Medicine, Charles University, Průmyslová 595, 25250 Vestec, Czech Republic

**Keywords:** apoptosis, caspase, intrinsic apoptosis, nanoparticles, nanotoxicity, oxidative stress

## Abstract

The therapeutic potential of redox-active nanoscale materials as antioxidant- or reactive oxygen species (ROS)-inducing agents was intensely studied. Herein, we demonstrate that the synthesized and characterized GdVO_4_:Eu^3+^ and LaVO_4_:Eu^3+^ nanoparticles, which have been already shown to have redox-active, anti-inflammatory, antibacterial, and wound healing properties, both in vitro and in vivo, worsen oxidative stress of L929 cells triggered by hydrogen peroxide or *tert*-butyl hydroperoxide (tBuOOH) at the concentrations that are safe for intact L929 cells. This effect was observed upon internalization of the investigated nanosized materials and is associated with the cleavage of caspase-3 and caspase-9 without recruitment of caspase-8. Such changes in the caspase cascade indicate activation of the intrinsic caspase-9-dependent mitochondrial but not the extrinsic death, receptor-mediated, and caspase-8-dependent apoptotic pathway. The GdVO_4_:Eu^3+^ and LaVO_4_:Eu^3+^ nanoparticle-induced apoptosis of oxidatively compromised L929 cells is mediated by ROS overgeneration, Ca^2+^ overload, endoplasmic reticulum stress-associated JNK (c-Jun N-terminal kinase), and DNA damage-inducible transcript 3 (DDIT3). Our findings demonstrate that GdVO_4_:Eu^3+^ and LaVO_4_:Eu^3+^ nanoparticles aggravate the oxidative stress-induced damage to L929 cells, indicating that they might potentially be applied as anti-cancer agents.

## 1. Introduction

Currently, cancer treatment still remains one of the major global challenges to healthcare. Cancer research has primarily focused on the development of novel therapeutic approaches and, over the recent years, the field has faced significant advances [1,2]. Fundamentally, cancer is considered to be a regulated cell death (RCD) disorder. In multiple types of cancer drivers, mutations affect genes, encoding the components of the signaling pathways to ensure escaping RCDs [3,4]. Apoptosis resistance in tumors frequently results in ineffective chemotherapy. This indicates that alternatives to apoptosis RCDs (necroptosis, ferroptosis, PANoptosis, pyroptosis, cuproptosis, etc.) can be targeted to guarantee the death of cancer cells [5]. It is important to note that tumors have also evolved mechanisms to evade these RCDs [6,7]. Notably, reactive oxygen species (ROS) are important regulators of cell death signaling. ROS can determine the cell fate in a concentration-dependent fashion not only by promoting cell death per se, but also by triggering a particular RCD mode, which might significantly affect the tumor microenvironment (TME) [8].

In cancer, redox metabolism is rewired, and malignant cells generally have higher basal ROS levels [9]. The ROS in cancer cells contribute to tumor promotion, progression and metastasis, cause DNA damage to increase the rate of novel mutations, and modify the cellular composition of the TME, making it pro-inflammatory [10,11]. However, the excessive generation of ROS results in inhibition of the proliferative signaling pathways, as well as the induction of apoptosis or ferroptosis. Thus, ROS act as a double-edged sword in cancer [11]. All these factors suggest that the application of redox active molecules is promising in anti-cancer therapy [12]. Multiple, frequently contradictory, ROS-targeting therapeutic strategies have been suggested for anti-cancer therapy, including application of antioxidants at early stages of cancer or for its prevention. Alternatively, induction of ROS elevation, as well as depletion of the antioxidant system can trigger death of tumor cells [13]. Notably, changes in the redox homeostasis of cancer cell might be helpful to guarantee selectivity of ROS targeting making the field promising for further research [10]. Of note, such selective cytotoxicity against tumor cells can be achieved by application of nanomaterials [14].

Advances in nanotechnology have resulted in the development of a myriad of redox active nanoscale materials, which can act as ROS scavengers or ROS generators [15]. Multiple nanoparticles (NPs) have been shown to generate ROS (e.g., via Fenton reaction). Alternatively, ROS production can be secondary, occurring in response to the impairment of the cellular metabolism mediated by NPs. In particular, NPs can cause mitochondrial dysfunction, which leads to excessive mitochondrial ROS (mitROS) production and the activation of intrinsic apoptosis [16]. Furthermore, nanoscale materials can be applied as drug delivery systems to ensure the targeted delivery of ROS-modulating agents [17,18] Nanotechnology-based ROS-mediating treatment approaches are applied in photodynamic and sonodynamic therapies [19]. Since the redox metabolism of tumor cells differs from non-cancerous cells, redox responsive NPs, which can be either pro-oxidant or antioxidant depending on the surrounding redox microenvironment, can switch to ROS generation in the pro-oxidant TME. In addition, these nanoscale materials can be used to enhance the effectiveness of oxidative stress-induced anti-cancer treatment, including radiation therapy (RT) [20].

In this study, we investigated LnVO_4_:Eu^3+^ (Ln = Gd or La) NPs, whose dual redox properties have been reported [21]. It is important to note that good biocompatibility of these NPs has been shown both in vitro and in vivo [22,23]. This encourages their further investigation as possible therapeutic agents. The rare-earth orthovanadate nanomaterial family has generated a significant interest in nanomedical research due to a wide array of properties and their potential applicability in different biomedical fields [24]. In particular, we have demonstrated that the LnVO_4_:Eu^3+^ NPs investigated in this study have antibacterial, anti-inflammatory, and wound healing effects [22,25,26].

In the current study, we further analyzed the cytotoxicity of LnVO_4_:Eu^3+^ NPs against L929 cells, a cell line widely used in nanotoxicological studies, to supplement the existing data on their biosafety [27]. In addition, we applied two exogenous oxidative stress models (H_2_O_2_- and *tert*-butyl hydroperoxide (tBuOOH)-induced oxidative damage) [28,29] to study the ability of rare-earth orthovanadate NPs to reinforce the ROS production and cell damage in impaired redox conditions, which can be further exploited in cancer therapy.

## 2. Results

### 2.1. Characterization of GdVO_4_:Eu^3+^ and LaVO_4_:Eu^3+^ Nanoparticles

**Figure 1:**
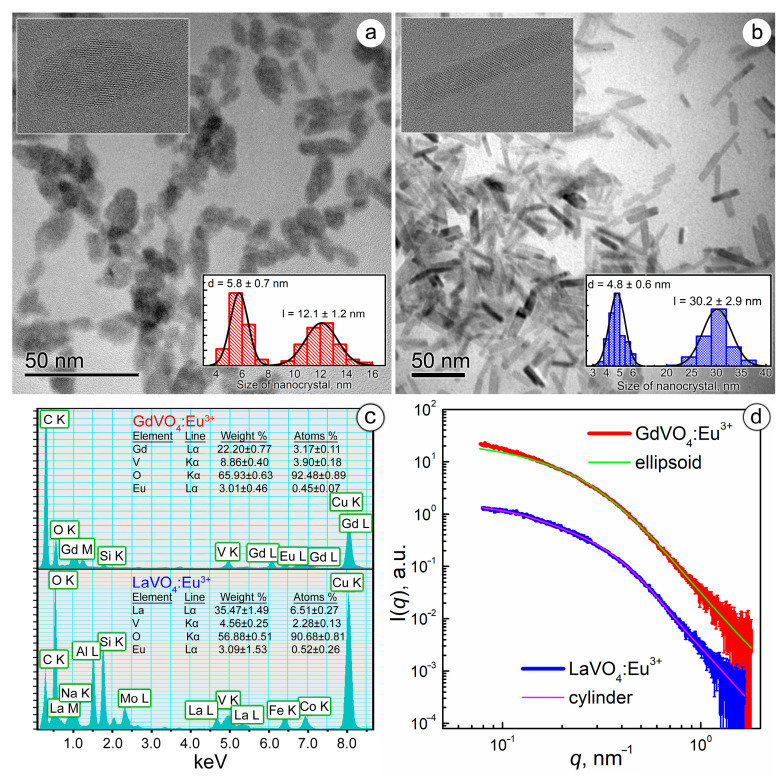
TEM images of the GdVO_4_:Eu^3+^ (Panel (**a**)) and LaVO_4_:Eu^3+^ (Panel (**b**)) nanoparticles (the insets show the size distribution diagrams and high-resolution micrographs of the synthesized nanoparticles). EDS spectra of the synthesized nanoparticles (Panel (**c**)). Experimental SAXS curves for the GdVO_4_:Eu^3+^ and LaVO_4_:Eu^3+^ nanosized materials from the as-prepared aqueous solutions (Panel (**d**)). The solid lines show the best fit to both the ellipsoidal and cylinder model.

The synthesized GdVO_4_:Eu^3+^ and LaVO_4_:Eu^3+^ NPs differed in size and shape. As shown in Figure 1a,b, the TEM images revealed that the GdVO_4_:Eu^3+^ NPs were ellipsoidal in shape with a semi-major axis (a) of 6 nm and a semi-minor axis (b) of 2.9 nm, which gave a characteristic length (l) of 12 nm and a diameter (d) of 5.8 nm. Meanwhile, the LaVO_4_:Eu^3+^ NPs were cylinders with d = 4.8 nm and *l* = 30.2 nm. High-resolution images, presented as insets in Figure 1a,b with pronounced lattice planes, confirmed the crystallinity of the LnVO_4_:Eu^3+^ NPs.

Electron scattering spectroscopy (EDS) analysis of the energy-dispersive X-ray spectra revealed that the chemical composition of the NPs corresponded to Gd_0.88_Eu_0.12_(VO_4_) and La_0.92_Eu_0.08_(VO_4_), respectively (Figure 1c).

The synthesized LnVO_4_:Eu^3+^ NPs were found to have a negative surface charge: the ζ-potential was −26.59 ± 0.71 mV for GdVO_4_:Eu^3+^ and −30.02 ± 0.91 mV for the LaVO_4_:Eu^3+^ NPs. High ζ-potential values pointed to a long-term stability of the obtained LnVO_4_:Eu^3+^ colloids, which aligned with our observations.

To evaluate the aggregation degree of the synthesized GdVO_4_:Eu^3+^ and LaVO_4_:Eu^3+^ NPs in aqueous solutions, which is critical for their biomedical applications, we analyzed SAXS data (Figure 1c) [30]. Experimental SAXS data were fitted by the model of two-axis ellipsoids (for GdVO_4_:Eu^3+^) and cylinders (for LaVO_4_:Eu^3+^) based on the findings obtained from electron microscopy (Figure 1d). The results of the SAXS data approximation were in good agreement with the TEM data, giving characteristic sizes for the GdVO_4_:Eu^3+^ (d = 6.8 nm and l = 16.3 nm) and LaVO_4_:Eu^3+^ (d = 6.5 nm and l = 30.0 nm) NPs, which points to the absence of NP aggregation in aqueous solutions.

### 2.2. L929 Cells Internalize GdVO_4_:Eu^3+^ and LaVO_4_:Eu^3+^ Nanoparticles

**Figure 2:**
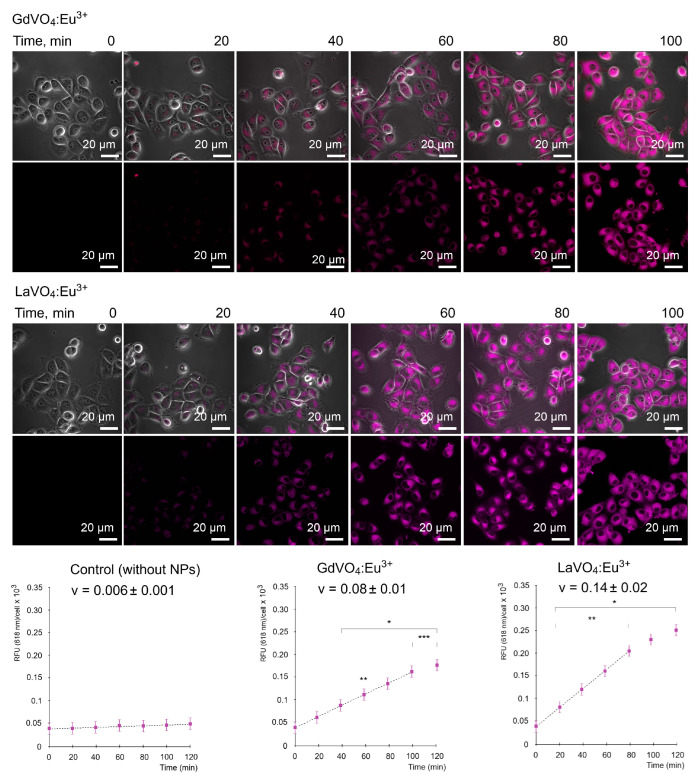
Representative LSCM images demonstrate the uptake of the GdVO_4_Eu^3+^ and LaVO_4_Eu^3+^ nanoparticles at 20 mg/L by the L929 cells. Internalization of the studies nanoscale materials was quantified (rfu per cell) and revealed a time-dependent pattern. The presented linear velocity (v) indicates the change in the fluorescence intensity of NPs over 1 minute and is calculated based on a linear range of curves (dashed line). ANOVA and post hoc Bonferroni tests were conducted, and the mean ± SD (*n* = 24) was determined. Note: * (*p* < 0.05); ** (*p* < 0.01); and *** (*p* < 0.001) were compared with the control samples.

As illustrated in Figure 2, the GdVO_4_:Eu^3+^ and LaVO_4_:Eu^3+^ NPs were capable of entering the L929 cells in a time-dependent manner. Importantly, the internalization of both NPs occurred in a comparable fashion and started after 40 min of incubation with the NPs at a concentration of 20 mg/L.

### 2.3. The GdVO_4_:Eu^3+^ and LaVO_4_:Eu^3+^ Nanoparticles Were Internalized by L929 Cells via Endocytosis

**Figure 3:**
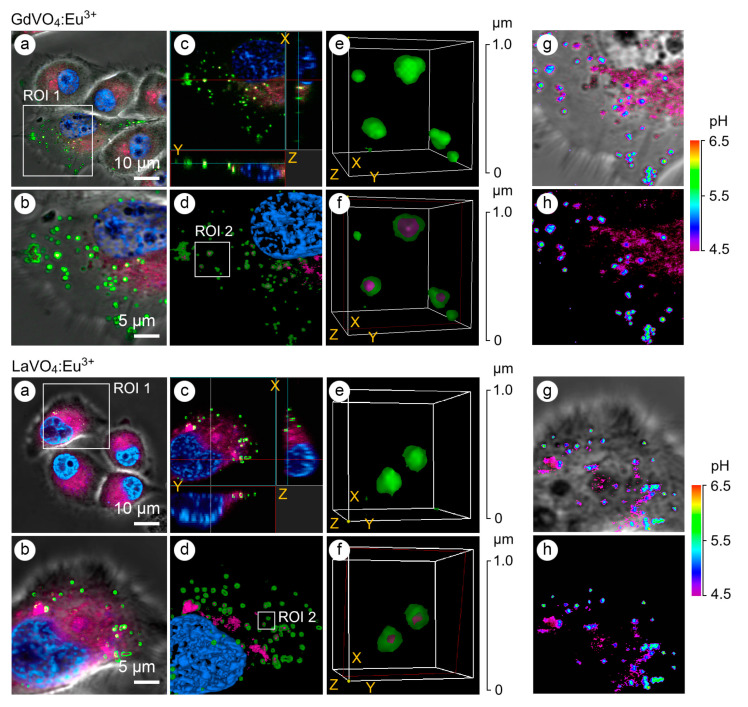
Representative LSCM images of the endosome-positive L929 cells following treatment with GdVO_4_Eu^3+^ and LaVO_4_Eu^3+^ nanoparticles at 20 mg/L for 1 h. The L929 cells were transfected with GFP-Rab5a/GFP-Rab7a and, 24 h after transfection, the cells were exposed to the investigated NPs. The Rab5a and Rab7a proteins were markers of the early and late endosomes, respectively. The GFP-Rab5a/GFP-Rab7a-positive endosomes (green) and NPs’ autofluorescence signals (magenta) were detected. The cell nuclei are shown in blue (DAPI staining). Single-optic section phase contrast and fluorescence-merged imaging (Panel (**a**)); magnified single-optic section fluorescence imaging of the ROI1 (Panel (**b**)); orthogonal XZ and YZ projections of the ROI1 (Panel (**c**)); 3D reconstruction of the ROI1 (Panel (**d**)); complete 3D reconstruction of the endosomes without and with the NPs’ fluorescence channel (magenta) of the ROI2 (Panel (**e**)); cross section of the endosomes (red frame) demonstrating the intra-endosomal localization of the NPs (magenta) of the ROI2 (Panel (**f**)); and pseudocolored and phase-contrast imaging of the endosomal pH using the LysoSensor ratiometric probe (molecular probes, L22460) of the ROI1 (Panels (**g**,**h**)). Ratiometric pseudocolored images were constructed from two emission images at 450 ± 33 nm and 510 ± 20 nm, respectively. Both were excited at 365 ± 8 nm. The cells were preliminarily exposed to pH calibration buffers (pH 4.5–6.5).

The application of early and late endosomal markers revealed that both vanadate NPs accumulated in the endosomes (Figure 3), suggesting that the internalization of the LnVO_4_:Eu^3+^ NPs occurred, at least partly, through endocytosis. In addition to the use of endosomal markers such as Rab5a and Rab7a, the type of endosomes was confirmed by determining the pH values in the vesicles since the early endosomes had a pH of 6.3, whereas the late endosomes had a pH of 5.5 (Figure 3). Furthermore, vesicles with a pH of 4.5 were also found to harbor the investigated NPs.

### 2.4. The GdVO_4_:Eu^3+^ and LaVO_4_:Eu^3+^ Nanoparticles Entered the Cell Nuclei and Increased Chromatin Condensation

**Figure 4:**
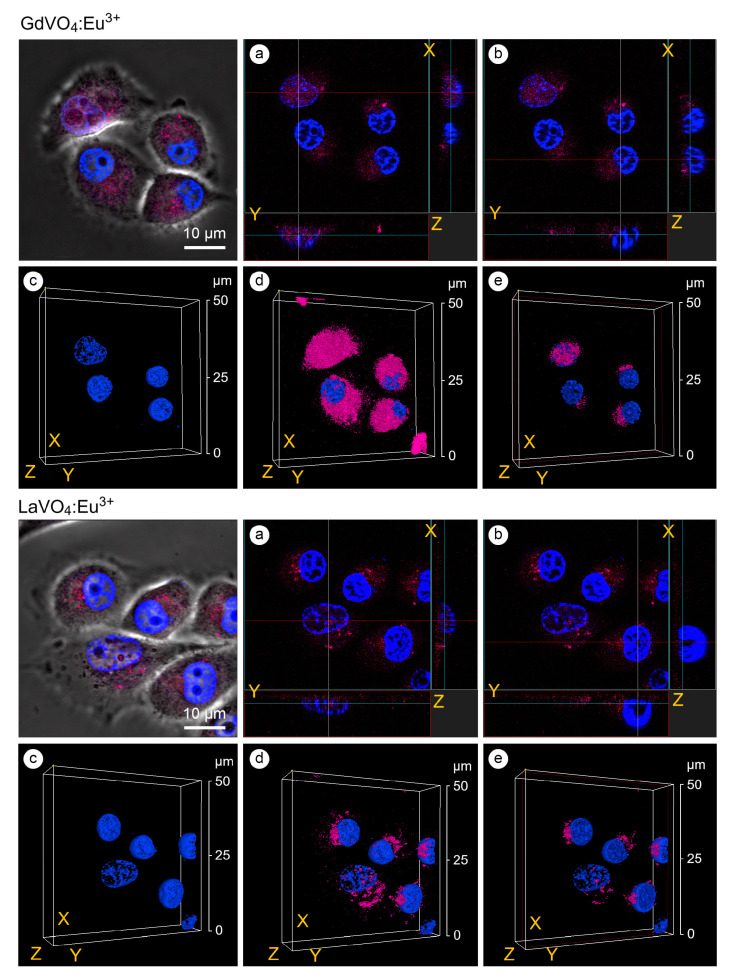
Confocal microscopy images with an orthogonal projection and 3D reconstruction of the L929 cells treated with the GdVO_4_Eu^3+^ and LaVO_4_Eu^3+^ nanoparticles at 20 mg/L for 60 min: orthogonal projection (white and red lines) of the cells with highly condensed (Chromatin Condensation Index, CCI = 17.2 ± 4.9) heterochromatin (Panel (**a**)); orthogonal projection (white and red lines) of the cells with regularly condensed (CCI = 6.8 ± 2.5) heterochromatin (Panel (**b**)); complete 3D reconstruction of the cells not showing (Panel (**c**)) and showing (Panel (**d**)) the channel of nanomaterials-dependent fluorescence (magenta); and cross section of the cell nuclei (red frame) (Panel (**e**)). Cell nuclei are shown in blue (DAPI staining).

As shown in Figure 4, the GdVO_4_:Eu^3+^ and LaVO_4_:Eu^3+^ NPs were accumulated around the nucleus and could penetrate inside. The presence of the investigated rare-earth orthovanadates in the cell nuclei was accompanied by an increase in the degree of chromatin condensation. Notably, its condensation was observed on the periphery of the nucleus (Figure 4).

### 2.5. The GdVO_4_:Eu^3+^ and LaVO_4_:Eu^3+^ Nanoparticles Did Not Promote Cell Death, Oxidative Stress, and the Elevation of Intracellular Calcium in the L929 Cells

**Figure 5:**
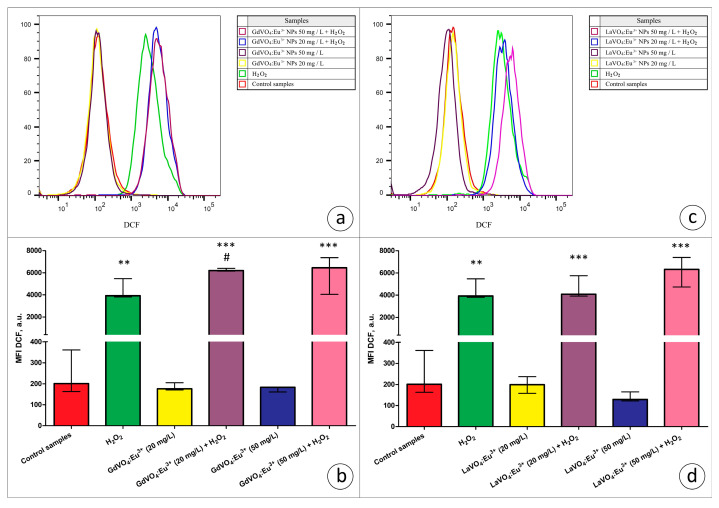
The rare-earth orthovanadate nanoparticles enhanced H_2_O_2_-mediated oxidative stress in the L929 cells. The GdVO_4_:Eu^3+^ nanoparticles increased the DCF-mediated fluorescence in the H_2_O_2_-treated samples (3 μM), indicating an increase in the ROS production in the L929 cells (Panels (**a**,**b**)). Likewise, the LaVO_4_:Eu^3+^ nanoparticles promoted H_2_O_2_-induced redox imbalance in the L929 cells (Panels (**c**,**d**)). At the same time, exposure of the L929 cells to both nanoscale materials used does not impair redox homeostasis. ANOVA and post hoc Bonferroni tests were conducted, and the Me and IQR (*n* = 3) were determined. Note: ** (*p* < 0.01); and *** (*p* < 0.001) were compared with the control samples. # (*p* < 0.05); were compared with the H_2_O_2_-treated samples.

**Figure 6:**
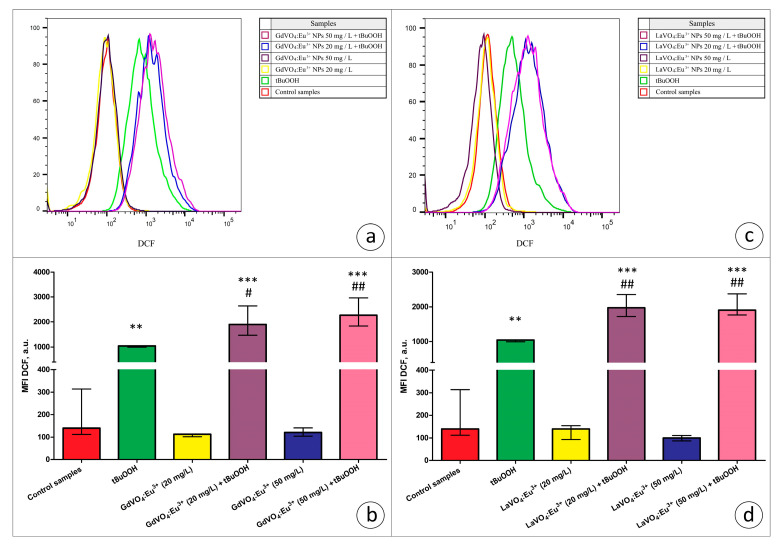
The GdVO_4_:Eu^3+^ and LaVO_4_:Eu^3+^ nanoparticles aggravated tBuOOH-induced impairment of redox homeostasis in the L929 cells. The GdVO_4_:Eu^3+^ (Panels (**a**,**b**)) and LaVO_4_:Eu^3+^ nanoparticles (Panels (**c**,**d**)) enhanced ROS generation in the L929 cells exposed to tBuOOH (0.5 mM). Data were statistically processed using ANOVA and post hoc Bonferroni tests, and the Me and IQR (*n* = 3) were determined. Note: ** (*p* < 0.01); and *** (*p* < 0.001) were compared with the control samples. # (*p* < 0.05); ## (*p* < 0.01) were compared with the tBuOOH-treated samples.

Viability-discriminating 7-aminoactinomycin D (7-AAD) staining revealed that the exposure of L929 cells to both rare-earth orthovanadate NPs at 20 and 50 mg/L did not affect the viability of the cells (data are not presented). Additionally, to assess the toxicity of the NPs, ROS production by viable L929 cells was estimated based on the dichlorofluorescein (DCF) fluorescence in the 7-AAD-negative cells. As illustrated in Figure 5 and Figure 6, the GdVO_4_:Eu^3+^ or LaVO_4_:Eu^3+^ NPs had no impact on the ROS-generating abilities of the L929 cells, indicating that, at the concentrations used, these particles did not promote redox imbalance.

**Figure 7:**
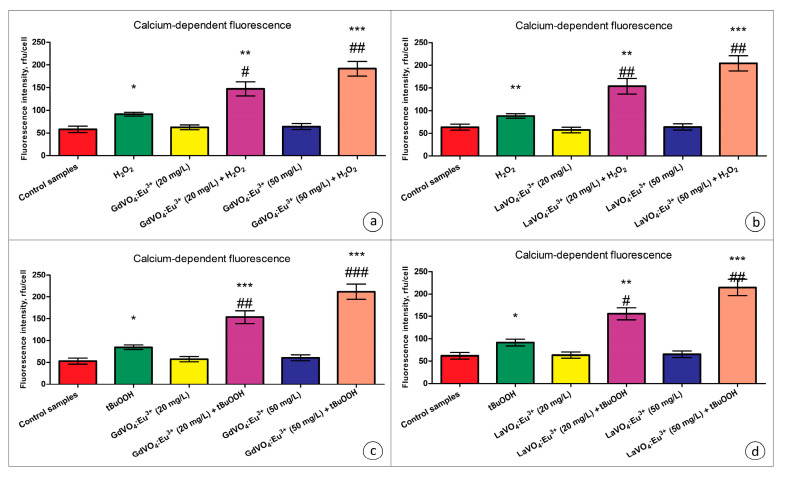
The GdVO_4_:Eu^3+^ and LaVO_4_:Eu^3+^ nanoparticles triggered Ca^2+^ elevation in the oxidatively damaged L929 cells. The GdVO_4_:Eu^3+^ (Panels (**a**,**c**)) and LaVO_4_:Eu^3+^ nanoparticles (Panels (**b**,**d**)) affected the calcium-specific fluorescence intensity in the L929 cells exposed to H_2_O_2_ and tBuOOH, respectively. ANOVA and Tukey’s tests were conducted, and the mean ± SEM (*n* = 3) were determined. Note: * (*p* < 0.05); ** (*p* < 0.01); and *** (*p* < 0.001) were compared with the control samples. # (*p* < 0.05); ## (*p* < 0.01); and ### (*p* < 0.001) were compared with the H_2_O_2_- or tBuOOH-treated samples. Rfu—relative fluorescence units.

As shown in Figure 7, the GdVO_4_:Eu^3+^ and LaVO_4_:Eu^3+^ NPs did not trigger elevation of the intracellular Ca^2+^ concentrations.

**Figure 8:**
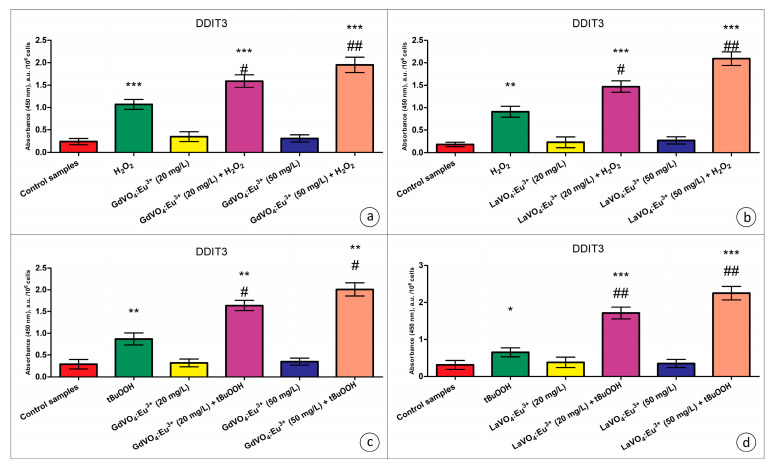
The GdVO_4_:Eu^3+^ and LaVO_4_:Eu^3+^ nanoparticle-induced damage to oxidatively stressed L929 cells was mediated by DDIT3. The GdVO_4_:Eu^3+^ (Panels (**a**,**c**)) and LaVO_4_:Eu^3+^ nanoparticles (Panels (**b**,**d**)) increased the DDIT3-specific absorbance in the L929 cells exposed to H_2_O_2_ and tBuOOH, respectively. ANOVA and Tukey’s tests were conducted, and the mean ± SEM (*n* = 3) were determined. Note: * (*p* < 0.05); ** (*p* < 0.01); and *** (*p* < 0.001) were compared with the control samples. # (*p* < 0.05); ## (*p* < 0.01) were compared with the H_2_O_2_- or tBuOOH-treated samples. A.u.—arbitrary units.

Additionally, no upregulation of total JNK (c-Jun N-terminal kinase), a pro-apoptotic regulatory kinase, was observed when the L929 cells were exposed to NPs (Table S1). Furthermore, JNK phosphorylation was not promoted by the investigated nanomaterials. Moreover, analysis of the content of DNA damage-inducible transcript 3 (DDIT3) (commonly referred to as C/EBP homologous protein (CHOP), which is a pro-apoptotic transcription factor) revealed that the GdVO_4_:Eu^3+^ and LaVO_4_:Eu^3+^ NPs at concentrations of 20 and 50 mg/L had no impact on its intracellular content (Figure 8).

### 2.6. The GdVO_4_:Eu^3+^ and LaVO_4_:Eu^3+^ Nanoparticles Trigger Neither Intrinsic nor Extrinsic Apoptosis in the Intact Murine L929 Cells

**Figure 9:**
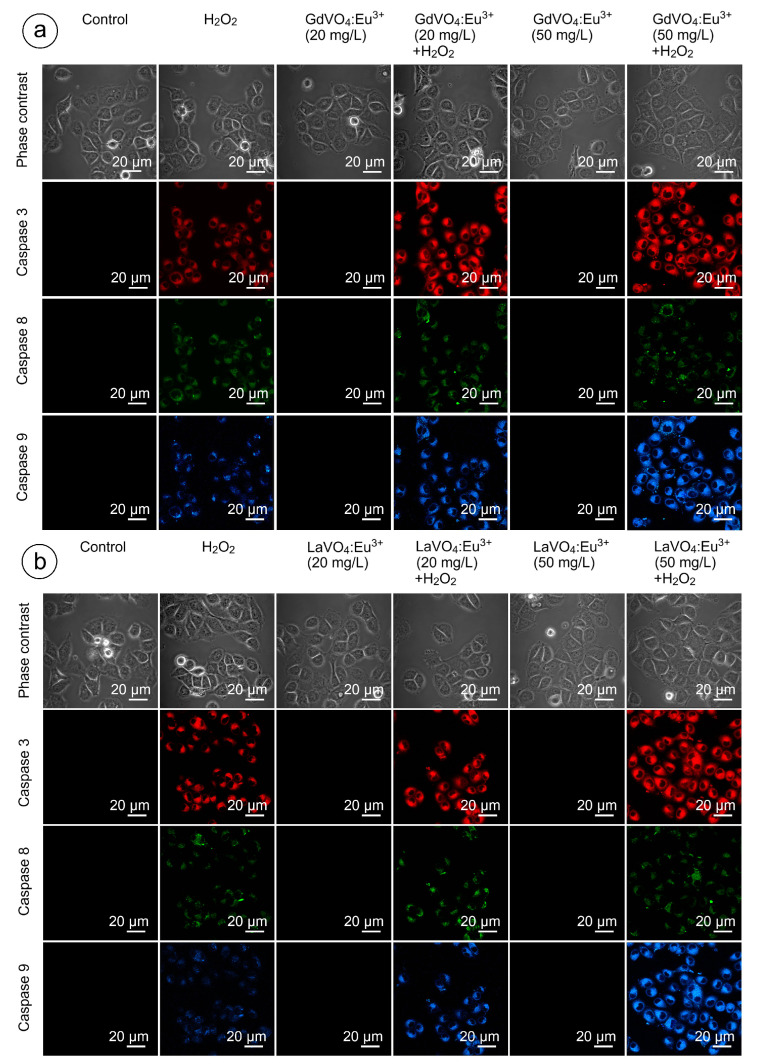
LSCM representative images of the caspase-3-, caspase-8-, and caspase-9-specific fluorescence in the L929 cells after incubation with the GdVO_4_Eu^3+^ (Panel (**a**)) or LaVO_4_Eu^3+^ (Panel (**b**)) nanoparticles in the presence of H_2_O_2_. Scale bar is 20 µm.

**Figure 10:**
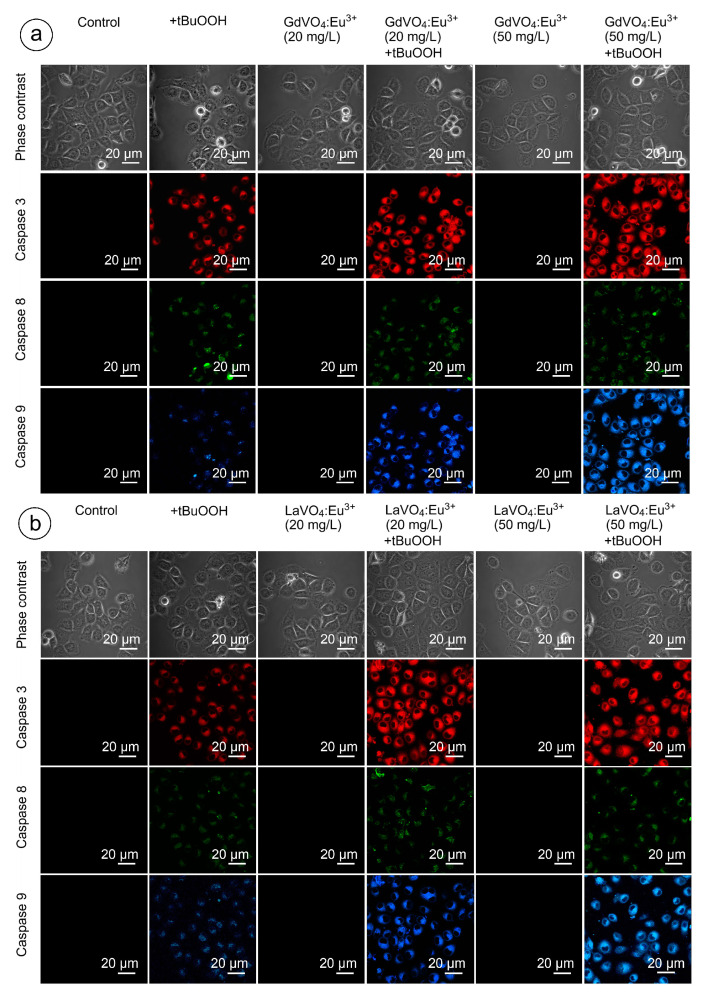
Representative confocal microscopy images show caspase-3-, caspase-8-, and caspase-9-specific fluorescence in tBuOOH-treated L929 cells exposed to GdVO_4_Eu^3+^ (panel (**a**)) or LaVO_4_Eu^3+^ (panel (**b**)) nanoparticles. Scale bar is 20 µm.

As shown in Figure 9 and Figure 10, and Table S2, none of the rare-earth orthovanadate NPs promoted activation of caspase-3, which is a key executioner caspase that is involved in both intrinsic and extrinsic pathways. Likewise, caspase-8 and caspase-9, which are crucial initiator caspases for extrinsic receptor-mediated and intrinsic mitochondrial apoptosis pathways, respectively, remained inactivated in the L929 cells exposed to NPs at 20 and 50 mg/L. These findings supplemented our data on the good safety profiles of the GdVO_4_:Eu^3+^ and LaVO_4_:Eu^3+^ NPs, indicating that these NPs did not promote apoptosis.

### 2.7. The GdVO_4_:Eu^3+^ and LaVO_4_:Eu^3+^ Nanoparticles Exacerbate Oxidative Stress in L929 Cells

In the present study, we used the models of H_2_O_2_- and tBuOOH-induced oxidative damage to assess the redox homeostasis-regulating properties of rare-earth orthovanadate NPs. Unexpectedly, both NPs were found to aggravate redox imbalance. This effect only reached statistical significance for the GdVO_4_:Eu^3+^ NPs at 20 mg/L in the case of H_2_O_2_-induced oxidative damage (Figure 1b and Figure 5a). At the same time, both NPs at 20 and 50 mg/L exacerbated tBuOOH-induced oxidative stress, evidenced by almost two-fold elevation of DCF-dependent fluorescence (Figure 6).

### 2.8. Ca^2+^ Signaling Contributes to the GdVO_4_:Eu^3+^ and LaVO_4_:Eu^3+^ Nanoparticle-Mediated Toxicity in Oxidatively Damaged Cells

Exposure of the oxidatively damaged L929 cells to the GdVO_4_:Eu^3+^ and LaVO_4_:Eu^3+^ NPs resulted in a dose-dependent increase (over two-fold) in the intracellular Ca^2+^ concentrations (Figure 7). The effect was observed for both types of the nanoscale materials used.

### 2.9. Orthovanadate Nanoparticles Accelerate Intrinsic, but Not Extrinsic Apoptosis in Oxidatively Damaged L929 Cells

Figure 9 and Figure 10 clearly demonstrate that H_2_O_2_- and tBuOOH-induced redox imbalance made the L929 cells more vulnerable to the LnVO_4_:Eu^3+^ NPs. Indeed, the quantification of data supported this conclusion (Table S2). The cleavage of caspase-3 following exposure of the H_2_O_2_- and tBuOOH-treated L929 cells to the NPs was observed to a higher degree compared to the oxidatively damaged non-exposed L929 cells, indicating that the GdVO_4_:Eu^3+^ and LaVO_4_:Eu^3+^ NPs enhanced oxidative stress-induced apoptosis, thereby facilitating cell death of the oxidatively compromised cells. Mostly, the fluorescent intensity of the corresponding cleaved caspase-3-dependent probe was over 2.5-fold higher. Importantly, caspase-8 activity was not affected following exposure to the GdVO_4_:Eu^3+^ and LaVO_4_:Eu^3+^ NPs in the H_2_O_2_- or tBuOOH-treated cells, which indicated that the rare-earth orthovanadate-induced apoptosis in oxidatively injured L929 cells was independent of death receptors. At the same time, the higher values of caspase-9-dependent fluorescence (over 2.5-fold) in the NP-treated cells exposed to H_2_O_2_ or tBuOOH suggested that the apoptosis induced by vanadates relied on the intrinsic mitochondrial pathway.

### 2.10. Exacerbation of Damage to the Oxidatively Stressed L929 Cells Is Mediated by JNK and DDIT3

As clearly demonstrated in Table S1, the dGdVO_4_:Eu^3+^ and LaVO_4_:Eu^3+^ NPs increased the JNK expression and the degree of its phosphorylation under alterations of redox homeostasis only, which indicated the activation of this regulatory kinase. In addition to JNK, DDIT3 is required to mediate the toxicity of the GdVO_4_:Eu^3+^ and LaVO_4_:Eu^3+^ NPs against oxidatively compromised L929 cells (Figure 8). Its expression was over 50% elevated following exposure to NPs in comparison with the cells undergoing oxidative stress.

## 3. Discussion

In this study, we evaluated the effects of the redox-active GdVO_4_:Eu^3+^ and LaVO_4_:Eu^3+^ NPs on intact and oxidatively damaged L929 cells to investigate the impact of changes in the redox homeostasis on the features of nano–bio interactions in the case of rare-earth orthovanadates. It is important to note that the GdVO_4_:Eu^3+^ and LaVO_4_:Eu^3+^ NPs were non-toxic to intact L929 cells at concentrations of at least 50 mg/L, which is in line with our earlier findings, which clearly demonstrated that LnVO_4_:Eu^3+^ NPs neither trigger eryptosis of erythrocytes nor alter the cell membrane of leukocytes [22,23,31]. Herein, we showed that the LnVO_4_:Eu^3+^ NPs did not reduce cell viability. In addition, neither ROS overgeneration, Ca^2+^ overload, nor apoptosis induction were observed. Notably, the cellular uptake of the NPs by L929 cells reported in this study for both GdVO_4_:Eu^3+^ and LaVO_4_:Eu^3+^ NPs at 20 mg/L did not provide the toxicity. Interestingly, these NPs showed different patterns of cellular uptake by erythrocytes. Erythrocytes were able to internalize the spherical GdVO_4_:Eu^3+^ NPs, but no uptake was observed for the cylindrical LaVO_4_:Eu^3+^ NPs [23]. Taken together, the LaVO_4_:Eu^3+^ NPs demonstrated the cell-type-dependent cellular uptake. There is strong evidence that endocytosis is a key pathway for the internalization of nanomaterials [32]. Indeed, our data show that NPs were found in the early and late endosomes. In this study, endocytosis of the GdVO_4_:Eu^3+^ and LaVO_4_:Eu^3+^ NPs was verified by application of the early and late endosome markers and determination of the pH values in the vesicles containing NPs. The early endosomes were characterized by higher pH values (pH 6.3) compared to the late endosomes (pH 5.5) [33]. Moreover, the NPs were found in the vesicles with a pH value of 4.5, which corresponds to the same pH of lysosomes. However, they were much smaller than the lysosomes and are most likely secretory vesicles of the Golgi apparatus containing acid hydrolases. They were linked with the subsequent formation of the lysosome after fusion of the late endosomes with secretory vesicles in the early response of the cells to xenobiotics [34].

Additionally, the cellular uptake of the LnVO_4_:Eu^3+^ NPs was followed by their nuclear uptake, which is associated with enhanced chromatin condensation. We believe that the findings we observed represent the mechanical response of the nucleus to external NP–nuclear membrane interactions. The similar effect was described as the mechanical response of nuclear chromatin to viral capsid–nuclear membrane interaction [35]. It is important to note that the nuclear integrity in response to mechanical stress is essential for maintaining cell survival. The accumulation of the LnVO_4_:Eu^3+^ NPs around the nucleus demonstrated in this study might indicate that they put pressure on the nuclear envelope. This might result in a higher chromatin stiffness and a softer nuclear lamina. Importantly, chromatin and lamin proteins are among the key factors determining nuclear mechanics [36]. Increased chromatin stiffness is associated with enhanced chromatin condensation, while lamina elasticity is mediated by A- and C-type lamin proteins [37,38]. Thus, the nuclear alterations observed in this study might be an adaptive response aimed at maintaining the nuclear morphology to reduce the mechanical stress on the nuclear membrane mediated by NPs. Therefore, the reported stiffness of NP-treated cell nuclei is supposed to be modulated by the attachment of GdVO_4_:Eu^3+^ and LaVO_4_:Eu^3+^ NPs to the nuclear pore complexes, leading to an increased nucleus permeability and nuclear uptake of NPs.

Unexpectedly, our findings suggest that despite the ROS-scavenging properties of the GdVO_4_:Eu^3+^ or LaVO_4_:Eu^3+^ NPs observed in the cell-free medium reported earlier [39], they can exacerbate the already existing redox imbalance in biological milieu. It has been demonstrated that oxidative stress-compromised cells are more prone to the toxicity of NPs [40,41,42]. In particular, deleterious effects of ROS are associated with mitochondrial dysfunction, which, in turn, contributes to the enhancement of oxidative stress via mitROS generation [43] since the mitochondrial electron transport chain is known to be the major ROS generator in cells [44]. It can be assumed that mitochondrial dysfunction and the structural damage to mitochondria induced by oxidants is worsened by the GdVO_4_:Eu^3+^ or LaVO_4_:Eu^3+^ NPs contributing to ROS overproduction and to the exacerbation of redox imbalance. Indeed, this is supported by the activation of the caspase-9 reported in this study. Caspase-9 is known to be crucial for the intrinsic mitochondrial apoptotic pathway [45]. Moreover, our previous findings demonstrate that LnVO_4_:Eu^3+^ NPs trigger the mitochondrial dysfunction in leukocytes at concentrations of 120 mg/L, thereby reducing the mitochondrial membrane potential (ΔΨm) of cells [25]. Furthermore, the NPs used in this study had a strikingly different impact on the ROS generation in nucleated mitochondria-containing leukocytes and enucleated mitochondria-lacking mature erythrocytes, suggesting that GdVO_4_:Eu^3+^ or LaVO_4_:Eu^3+^ NPs mainly affect mitROS production [46]. The ROS production promoted by the NPs resulted in the activation of apoptosis in the oxidatively damaged L929 cells. Notably, the extrinsic death receptor-associated pathway was not involved in the GdVO_4_:Eu^3+^ or LaVO_4_:Eu^3+^ NP-mediated toxicity in contrast to the intrinsic pathway, which highlights the importance of the mitochondrial dysfunction. The extrinsic pathway is known to be mediated by the stimulation of death receptors like Fas, tumor necrosis factor receptors (TNF-Rs), TNF-related apoptosis-inducing ligand receptors (TRAIL-Rs), etc. [47]. Accumulating evidence suggests that NPs can trigger both intrinsic and extrinsic apoptosis, such as, for instance, nano-copper-triggered, Fas-dependent apoptosis [48]. Likewise, AuNPs promote Fas externalization and caspase-8 activation [49]. However, the LnVO_4_:Eu^3+^ NPs were incapable of inducing the extrinsic apoptosis in the current study probably due to the inability of these NPs to act as ligands for death receptors.

Ca^2+^ signaling plays an important role in cell death signaling, and Ca^2+^ overload triggers the intrinsic apoptotic pathway [50,51,52]. In this study, the Ca^2+^ accumulation in the cells was shown to contribute to the GdVO_4_:Eu^3+^ or LaVO_4_:Eu^3+^ NP-mediated toxicity against oxidatively stressed L929 cells only. In general, the cytotoxicity of multiple NPs was associated with the interplay between the Ca^2+^ overload and oxidative stress. For instance, the Ca^2+^-dependent cell death of the human epithelial cells exposed to ZnO NPs can be mitigated by ROS scavengers by reducing the Ca^2+^ overload [53]. Alternatively, AgNPs have been shown to trigger ROS overproduction in neutrophils by increasing the content of intracellular Ca^2+^ [54]. Thus, more studies are required to deepen our knowledge of the crosstalk between ROS and Ca^2+^ signaling in nanomaterial-induced cytotoxicity. Additionally, analysis of the pro-apoptotic factors involved in the regulation of mitochondria-associated apoptosis, namely JNK and DDIT3, corroborated our conclusions on the importance of the intrinsic apoptotic pathway. Severe endoplasmic reticulum (ER) stress results in the activation of both these enzymes. JNK and DDIT3 are known to reduce the anti-apoptotic effect of Bcl-2. DDIT3 (CHOP) downregulates anti-apoptotic Bcl-2, while JNK regulates its activity primarily via phosphorylation. Additionally, JNK phosphorylates pro-apoptotic Bim with the consecutive activation of Bax and Bak [55,56]. In turn, Bax and Bak promote the mitochondrial outer membrane permeabilization (MOMP) associated with the release of cytochrome c, its binding to apoptotic protease activating factor 1 (Apaf-1), and the downstream activation of the caspase cascade [57].

Our observations might open up novel therapeutic avenues for the GdVO_4_:Eu^3+^ or LaVO_4_:Eu^3+^ NPs. Radiation therapy (RT) is the mainstream approach in the management of cancer, and its mechanisms are widely associated with excessive ROS generation [58]. However, radiation-induced tissue damage remains one of the major challenges of this valuable tool when used in the therapy of neoplasms [59,60]. NPs have been shown to increase the therapeutic window of RT and immunotherapy [61]. Our study paves the way for the application of rare-earth orthovanadate nanoparticles as selective ROS-upregulating agents for enhancing the tumoricidal effects of RT and precluding damage to non-tumor tissues. The selective enhancement of ROS generation in the cells with compromised redox homeostasis against the background of low toxicity against the normal redox-balanced cells observed in this study suggests that it is promising to further explore the potential therapeutic implications of GdVO_4_:Eu^3+^ NPs or LaVO_4_:Eu^3+^ NPs as anti-cancer agents.

To sum up, we suggest the implications of oxidative stress-exacerbating GdVO_4_:Eu^3+^ or LaVO_4_:Eu^3+^ NPs as a strategy to improve the effectiveness of RT in cancer treatment. Herein, these nanoparticles show selective exacerbation of oxidative stress and activation of the intrinsic apoptosis in oxidatively damaged cells.

## 4. Materials and Methods

### 4.1. Chemicals

Gadolinium chloride hexahydrate (GdCl_3_·6H_2_O, 99.9%), lanthanum chloride heptahydrate (LaCl_3_·6H_2_O, 99.9%), europium chloride hexahydrate (EuCl_3_·6H_2_O, 99.9%), anhydrous sodium metavanadate (NaVO_3_, 96%), and trisodium citrate dihydrate (Na_3_C_6_H_5_O_7_, 99%) were purchased from Acros Organics (Waltham, MA, USA) and used as received.

### 4.2. LnVO_4_:Eu^3+^(Ln = Gd, La) Synthesis and Characterization

The two types of LnVO_4_:Eu^3+^ NPs, which differ in size due to the presence of different cations, Gd and La, were synthesized for this study in the form of water colloids. The colloidal solutions of the Gd_0.88_Eu_0.12_VO_4_ and La_0.92_Eu_0.08_VO_4_ NPs were synthesized using a co-precipitation method that has been described earlier [39,46]. Briefly, 50 mL of aqueous solutions containing 9 mmol/L of GdCl_3_·6H_2_O (for GdVO_4_:Eu^3+^ NPs) or 9 mmol/L of LaCl_3_·6H_2_O (for LaVO_4_:Eu^3+^ NPs) and 1 mmol/L of EuCl_3_·6H_2_O were prepared and mixed with 37.5 mL of Na_3_C_6_H_5_O_7_. Thereafter, 8 mL of a 0.01 mol/L Na_3_VO_4_ solution was added dropwise to the mixture, adjusting the pH to 10.5. The mixture was then vigorously stirred using a magnetic stirrer until a transparent solution was formed. This solution was refluxed for 16 h in a round-bottom flask, forming transparent colloidal solutions of either of Gd_0.88_Eu_0.12_VO_4_ and La_0.92_Eu_0.08_VO_4_ NPs. At the next stage, the solutions were dialyzed using a cellulose dialysis membrane with a nominal molecular weight cutoff of 3.5 kDa (CelluSepH1, Advion Interchim Scientific, Montluçon, France) for 24 h against deionized water, with three water changes every 8 h.

Electron microscopy images of the synthesized LnVO_4_:Eu^3+^ NPs were obtained by Transmission Electron Microscopy (TEM, JEM-1230 Transmission Electron Microscope, JEOL, Akishima, Tokyo, Japan). The chemical composition of the nanoparticles was determined using energy-dispersive X-ray spectroscopy with an EDS detector (resolution = 138 eV, detectable Z ≥ B).

The surface charge for the synthesized LnVO_4_:Eu^3+^ NPs was evaluated using a ZetaPALS/BI-MAS analyzer (Brookhaven Instruments Corp., Nashua, NH, USA), which was operated in the phase analysis light scattering mode at 25 °C.

The small-angle X-ray scattering (SAXS) method was applied to estimate the size and aggregation degree of the synthesized LnVO_4_:Eu^3+^ NPs. SAXS measurements were conducted using a Nano-inXider instrument (Xenocs, Grenoble, France) configured for beamstopless high-resolution analysis. The solutions under investigation were encapsulated within 1.5 mm borosilicate capillaries (Hilgenberg, Malsfeld, Germany). Each two-dimensional SAXS image was acquired over 20 min, followed by standard data processing, incorporating corrections for background noise, detector pixel sensitivity, and instrument parameters. The small-angle scattering data were fitted to the cylinder and 2-axis ellipsoid form factors using SasView software (v.6.0.0).

### 4.3. Cell Line and Incubation Conditions

The murine fibroblast L929 cells were kindly provided by the Institute for Problems of Cryobiology and Cryomedicine of the National Academy of Sciences of Ukraine, and they were thawed from three different vials and cultured in a Dulbecco’s Modified Eagle’s Medium (DMEM, BioWest, Nuaillé, France) containing 10% fetal bovine serum (FBS, BioWest, Nuaillé, France) and 100 U/mL of penicillin/streptomycin (Thermo Fischer Scientific, Waltham, MA, USA) in 25 cm^2^ culture flasks (SPL, Pyeongtaek, Republic of Korea) that were placed in a CO_2_ incubator at optimal growing temperature conditions (i.e., 37 °C and 5% CO_2_).

For the confocal microscopy-based experiments, the mouse L929 fibroblast cells were cultured in DMEM supplemented with 10% FBS, 100 U/mL of penicillin, 100 μg/mL of streptomycin, and 1 mM of sodium pyruvate under a humidified atmosphere of 37 °C and 5% CO_2_ using a Galaxy 14S incubator (Eppendorf, Hamburg, Germany). The day before the experiment, the L929 cells were seeded directly onto 35 mm confocal dishes (VWR, 75856-742) to ensure 86–89% confluency on the day of the experiment.

### 4.4. Internalization of Nanoparticles

The photoluminescence excitation and emission properties of the GdVO_4_:Eu^3+^ or LaVO_4_:Eu^3+^ NPs in the UV-A and visible regions (350–700 nm) were acquired by a Lumina Fluorescence Spectrometer (Thermo Fisher Scientific, Waltham, MA, USA). The excitation spectrum showed an intense absorption band in the UV-A region at 390 nm. Excitation resulted in the appearance of a strong narrow red emission at 618 nm [62]. Briefly, the internalization of the GdVO_4_:Eu^3+^ or LaVO_4_:Eu^3+^ NPs were evaluated by adding NPs to the culture medium at a concentration of 20 mg/L, with further measurements of the relative fluorescence being emitted at 0, 20, 40, 60, 80, and 100 min. Prior to imaging, the culture medium with GdVO_4_:Eu^3+^ or LaVO_4_:Eu^3+^ NPs was removed and replaced with a pre-warmed live-cell imaging solution (Life Technologies, Carlsbad, CA, USA). The images of the live cells were captured by confocal microscopy at an excitation of 391 nm and an emission of 618 nm. Cellular uptake was calculated in the linear region of the fluorescence intensity curves.

### 4.5. ROS Detection

The cells were harvested by trypsinization (0.25% trypsin-EDTA, Thermo Fischer Scientific, Waltham, MA, USA) and resuspended in 1 mL of the complete medium described above at 10 × 10^5^ cells per ml. To detect the ability of the NPs in affecting the redox processes in cells, the L929 cells were incubated with H_2_O_2_ (3 μM) and either GdVO_4_:Eu^3+^ NPs or LaVO_4_:Eu^3+^ NPs at concentrations of 20 or 50 mg/L for 15 min. Additionally, the model of tBuOOH-induced alterations of redox homeostasis was used to determine the redox-regulating effects of NPs. Briefly, GdVO_4_:Eu^3+^ NPs or LaVO_4_:Eu^3+^ NPs at concentrations of 20 or 50 mg/L were incubated with L929 fibroblasts in the presence of tBuOOH (0.5 mM) for 1 h. The optimal concentrations of oxidative stress-inducing agents were determined using a wide range of different concentrations of both agents in a preliminary experiment.

Following incubation, the cells were stained with 7-AAD and 2′,7′-dichlorodihydrofluorescein diacetate (H2DCFDA). Briefly, the cells were washed twice with phosphate-buffered saline (PBS; BD Biosciences, Franklin Lakes, NJ, USA), which were stained with 5 μL of 7-AAD (BD Pharmingen^TM^, BD Biosciences, Franklin Lakes, NJ, USA) and 5 μM of H2DCFDA (Invitrogen^TM^; Waltham, MA, USA). The suspensions were incubated in the dark for 30 min. Fluorescence was detected by a BD FACS Canto II flow cytometer (Becton Dickinson, Franklin Lakes, NJ, USA). DCF fluorescence was detected in viable 7-AAD-negative L929 cells. FlowJo (v10; BD Biosciences, Franklin Lakes, NJ, USA) was used to process the flow cytometry data and to prepare figures.

### 4.6. Calcium Assay

Intracellular calcium concentrations were directly detected using a Fura 2-AM probe (Abcam, ab120873, Cambridge, UK) in accordance with the manufacturer’s recommendations. To stain the L929 cells with the Fura 2-AM probe, an appropriate aliquot of its stock solution in DMSO (Invitrogen, D12345, Waltham, MA, USA) was added to 0.25 mL of DPBS buffer (Gibco, 14190144, Waltham, MA, USA). After vortexing (IKA TTS3 shaker, 4 s), the solution was added to fibroblast monolayers in a 6-well plate (Cellvis, NC0452316, Mountain View, CA, USA) to obtain a final probe concentration of 10 μM (<0.25% DMSO). Before acquiring the fluorescence data, the L929 monolayers were incubated with the probe for 1 h at 37 °C in the dark. Determination of the content of calcium in the cells was carried out by fluorometry (excitation 350 nm/emission 505 nm) on a FL600 microplate multimodal reader (BioTek, Winooski, VT, USA).

### 4.7. Determination of Total and Phosphorylated JNK

The content of the total and phosphorylated JNK in the lysate of the L929 cells was determined after incubation with NPs that were immunoenzymatically based on the spectrophotometric detection (450 nm) using a FL600 microplate multimodal reader (BioTek, Winooski, VT, USA) with the help of a RayBio^®^ Phospho-JNK (T183/Y185) and Total JNK ELISA Kit (RayBiotech, PEL-JNK-T183-T-1, Winooski, VT, USA). NP-40 buffer (Thermo Scientific, Waltham, MA, USA, cat. no. J60766.AP) was used for cell lysis.

### 4.8. DDIT3 Detection

The DDIT3 protein in the lysate of the L929 cells was analyzed after incubation with GdVO_4_:Eu^3+^ and LaVO_4_:Eu^3+^ NPs using a Mouse DDIT3/CHOP ELISA kit (LS Bio, LS-F9101, Lynnwood, WA, USA). A FL600 microplate multimodal reader (BioTek, Winooski, VT, USA) was used to acquire spectrophotometric data at 450 nm. The cells were lysed as outlined above.

### 4.9. Detection of Caspases

A Caspase Multiplex Activity Assay Fluorometric Kit (Abcam, ab219915, Cambridge, UK) was used to detect the cleaved caspase-3, caspase-8, and caspase-9. It is based on the application of DEVD-ProRed, IETD-R110, and LEHD-AMC as fluorogenic indicators for caspase-3, caspase-8, and caspase-9, respectively. When caspase is cleaved, the corresponding fluorochrome is released: ProRed™ (red fluorescence), R110™ (green fluorescence), and AMC™ (blue fluorescence), respectively. Their fluorescence is proportional to the caspase activity.

Briefly, the DEVD-ProRed, IETD-R110, and LEHD-AMC reagents were added to 0.5 mL of pre-warmed (37 °C) assay buffer. The mixture was vortexed (IKA TTS3 shaker, 4 s) and transferred to L929 monolayers cultured in a 35 mm single well. The final probe concentration was 0.01 μM. Incubation was performed for 30 min at 37 °C in the dark.

Confocal microscopy-based detection of the activity was carried out at an excitation of 535 nm/emission of 620 nm (caspase-3), an excitation of 650 nm/emission of 670 nm (caspase-8), and an excitation of 370 nm/emission of 450 nm (caspase 9).

### 4.10. Confocal Microscopy

The cells were directly visualized on a laser scanning confocal microscope FV10i-LIV (Olympus, Tokyo, Japan) equipped with a 60/1.2 NA water immersion objective and a system of intravital cell incubation (37.0 ± 0.1 °C, 5.0 ± 0.1% CO_2_, 99.5 ± 0.1% RH) in 35 mm confocal dishes (VWR, 75856-742). The images were acquired with a scanning mode format of 1024 × 1024 pixels. The pinhole aperture was 1 Airy unit. The confocal images shown are representative images of 10 fields of view (15 cells per field of view were scored). The 3D imaging dataset was assembled from 50 optical sections in the regions of interest. Post-rendering of the obtained images including the cells’ autofluorescence subtraction and measurements of fluorescence intensities were performed using Olympus cellSens 4.2 software (Olympus licensed).

### 4.11. Statistical Analysis

Due to a comparison of multiple independent variables, one-way analysis of variance (ANOVA) and post hoc Bonferroni or Tukey’s tests were used to statistically process the obtained numerical data using Graph Pad Prism 5.0 software (USA). The difference was considered statistically significant at *p* < 0.05.

## 5. Conclusions

Our findings indicate that the redox-active GdVO_4_:Eu^3+^ or LaVO_4_:Eu^3+^ nanoparticles can enhance damage to oxidatively stressed L929 cells at concentrations that are non-toxic to normal L929 cells. The effects are associated with the internalization of the investigated nanoscale materials and are mediated by the induction of the intrinsic mitochondrial apoptotic pathway, ROS overproduction, Ca^2+^ signaling, and the recruitment of ER stress-associated JNK and DDIT3. These results might pave the way for further investigation of the GdVO_4_:Eu^3+^ or LaVO_4_:Eu^3+^ nanoparticles as anti-cancer agents.

## Data Availability

The data that support the findings of this study were generated at V.N. Karazin Kharkiv National University, the Institute for Scintillation Materials of the National Academy of Sciences of Ukraine, the Institute for Problems of Cryobiology and Cryomedicine of the National Academy of Sciences of Ukraine, and the Kharkiv National Medical University. These data are available from the corresponding authors upon reasonable request.

## Supplementary Materials

The following supporting information can be downloaded at: https://www.mdpi.com/article/10.3390/ijms252111687/s1.
