# Supplementary material for: GdVO4:Eu3+ and LaVO4:Eu3+ Nanoparticles Exacerbate Oxidative Stress in L929 Cells: Potential Implications for Cancer Therapy
Source: Int J Mol Sci. 2024 Oct 30;25(21):11687. doi: 10.3390/ijms252111687 (PMC11546343; doi:10.3390/ijms252111687)
Supplement: Supplementary file 1 [file ijms-25-11687-s001.zip › Table S1.pdf]

Table S1. Total JNK and Phospho-JNK (Thr183/Tyr185)-specific absorbance (450 nm) in the lysate of L929 cells treated exposed to $GdVO_4:Eu^{3+}$ and $LaVO_4:Eu^{3+}$ nanoparticles.

Groups	Absorbance (450 nm), a.u. /10^6 cells		Total JNK/Phospho-JNK ratio
	Total JNK	Phospho-JNK	
Control	0.38 ± 0.10	0.15 ± 0.08	2.53
H_2O_2	0.87 ± 0.16*	0.51 ± 0.12**	1.70
$GdVO_4:Eu^{3+}$ (20 mg/L)	0.35 ± 0.14	0.13 ± 0.05	2.69
$GdVO_4:Eu^{3+}$ (20 mg/L) + H_2O_2	1.43± 0.17**; #	1.24 ± 0.15***; #	1.15
$GdVO_4:Eu^{3+}$ (50 mg/L)	0.32 ± 0.12	0.11 ± 0.06	2.91
$GdVO_4:Eu^{3+}$ (50 mg/L) + H_2O_2	1.97 ± 0.17***; #	1.76 ± 0.15***; ##	1.12
	Absorbance (450 nm), a.u. /10^6 cells		Total JNK/Phospho-JNK ratio
	Total JNK	Phospho-JNK	
Control	0.42 ± 0.11	0.17 ± 0.08	2.47
H_2O_2	0.92 ± 0.15*	0.67 ± 0.15**	1.37
$LaVO_4:Eu^{3+}$ (20 mg/L)	0.38 ± 0.12	0.15 ± 0.07	2.53
$LaVO_4:Eu^{3+}$ (20 mg/L) + H_2O_2	1.65± 0.18**; #	1.49 ± 0.15**; #	1.10
$LaVO_4:Eu^{3+}$ (50 mg/L)	0.35 ± 0.15	0.15 ± 0.10	2.33
$LaVO_4:Eu^{3+}$ (50 mg/L) + H_2O_2	2.11 ± 0.18***; #	1.93 ± 0.16***; ##	1.09
	Absorbance (450 nm), a.u. /10^6 cells		Total JNK/Phospho-JNK ratio
	Total JNK	Phospho-JNK	
Control	0.32 ± 0.07	0.11 ± 0.05	2.90
tBOOH	0.81 ± 0.14*	0.63 ± 0.15***	1.28
$GdVO_4:Eu^{3+}$ (20 mg/L)	0.41 ± 0.16	0.15 ± 0.12	2.73
$GdVO_4:Eu^{3+}$ (20 mg/L) + tBOOH	1.57± 0.15**; #	1.39 ± 0.12***; #	1.13
$GdVO_4:Eu^{3+}$ (50 mg/L)	0.44 ± 0.13	0.16 ± 0.08	2.75
$GdVO_4:Eu^{3+}$ (50 mg/L) + tBOOH	2.05 ± 0.18**; ##	1.87 ± 0.14***; ##	1.09
	Absorbance (450 nm), a.u. /10^6 cells		Total JNK/Phospho-JNK ratio
	Total JNK	Phospho-JNK	
Control	0.51 ± 0.10	0.19 ± 0.08	2.68
tBOOH	1.06 ± 0.13*	0.82 ± 0.15**	1.29
$LaVO_4:Eu^{3+}$ (20 mg/L)	0.55 ± 0.14	0.18 ± 0.10	3.05
$LaVO_4:Eu^{3+}$ (20 mg/L) + tBOOH	1.71± 0.17***; #	1.56 ± 0.13**; #	1.09
$LaVO_4:Eu^{3+}$ (50 mg/L)	0.47 ± 0.12	0.17 ± 0.11	2.76
$LaVO_4:Eu^{3+}$ (50 mg/L) + tBOOH	2.15 ± 0.15***; #	1.98 ± 0.15***; ##	1.08

Note: ANOVA and Tukey's tests, mean ± SEM (n = 3). Note: * ($p < 0.05$); ** ($p < 0.01$); *** ($p < 0.001$) compared with the control samples; # ($p < 0.05$); ## ($p < 0.01$); ### ($p < 0.001$) compared with the H_2O_2- or tBOOH-treated samples. A.u. – arbitrary units.
