# Supplementary material for: GdVO4:Eu3+ and LaVO4:Eu3+ Nanoparticles Exacerbate Oxidative Stress in L929 Cells: Potential Implications for Cancer Therapy
Source: Int J Mol Sci. 2024 Oct 30;25(21):11687. doi: 10.3390/ijms252111687 (PMC11546343; doi:10.3390/ijms252111687)
Supplement: Supplementary file 1 [file ijms-25-11687-s001.zip › Table S2.pdf]

Table S2. The single cell caspases-specific fluorescence intensity in L929 cells exposed to $GdVO_4:Eu^{3+}$ and $LaVO_4:Eu^{3+}$ nanoparticles.

Groups	Fluorescence intensity, rfu/cell		
	Caspase-3	Caspase-8	Caspase-9
Control	4.1 ± 1.5	4.7 ± 1.9	5.6 ± 2.1
H_2O_2	70.2 ± 11.5***	53.6 ± 8.8***	59.1 ± 10.3***
$GdVO_4:Eu^{3+}$ (20 mg/L)	6.6 ± 2.9	6.1 ± 1.8	8.7 ± 2.5
$GdVO_4:Eu^{3+}$ (20 mg/L) + H_2O_2	128.5 ± 16.9***; #	49.2 ± 11.7***	114.8 ± 15.1***; #
$GdVO_4:Eu^{3+}$ (50 mg/L)	8.3 ± 3.7	7.7 ± 3.5	9.5 ± 3.2
$GdVO_4:Eu^{3+}$ (50 mg/L) + H_2O_2	179.1 ± 15.3***; ##	57.4 ± 13.9***	161.7 ± 17.5***; ##
	Fluorescence intensity, rfu/cell		
	Caspase-3	Caspase-8	Caspase-9
Control	6.4 ± 1.9	4.2 ± 1.7	6.1 ± 2.3
H_2O_2	59.5 ± 9.3***	61.4 ± 11.2***	67.9 ± 12.5***
$LaVO_4:Eu^{3+}$ (20 mg/L)	7.1 ± 1.5	5.8 ± 2.1	7.9 ± 2.2
$LaVO_4:Eu^{3+}$ (20 mg/L) + H_2O_2	131.9 ± 14.6***; #	57.7 ± 9.4***	128.1 ± 16.5***; #
$LaVO_4:Eu^{3+}$ (50 mg/L)	7.5 ± 2.3	7.9 ± 2.2	8.1 ± 3.5
$LaVO_4:Eu^{3+}$ (50 mg/L) + H_2O_2	183.5 ± 17.1***; ##	63.1 ± 10.4***	177.9 ± 15.4***; ##
	Fluorescence intensity, rfu/cell		
	Caspase-3	Caspase-8	Caspase-9
Control	4.8 ± 1.6	5.2 ± 1.9	6.4 ± 2.5
tBOOH	61.1 ± 8.5***	58.9 ± 10.1***	57.3 ± 12.7***
$GdVO_4:Eu^{3+}$ (20 mg/L)	6.3 ± 1.9	6.8 ± 2.5	7.5 ± 2.9
$GdVO_4:Eu^{3+}$ (20 mg/L) + tBOOH	124.1 ± 12.0***; #	53.8 ± 10.5***	118.7 ± 13.6***; #
$GdVO_4:Eu^{3+}$ (50 mg/L)	8.1 ± 3.3	7.5 ± 2.8	8.0 ± 3.1
$GdVO_4:Eu^{3+}$ (50 mg/L) + tBOOH	155.8 ± 14.3***; ##	60.9 ± 11.8***	173.1 ± 14.7***; #
	Fluorescence intensity, rfu/cell		
	Caspase-3	Caspase-8	Caspase-9
Control	6.5 ± 2.7	7.1 ± 2.9	6.8 ± 2.3
tBOOH	73.1 ± 11.8***	51.1 ± 8.5***	77.1 ± 11.0***
$LaVO_4:Eu^{3+}$ (20 mg/L)	8.1 ± 3.7	7.0 ± 2.5	8.4 ± 2.7
$LaVO_4:Eu^{3+}$ (20 mg/L) + tBOOH	129.5 ± 15.3***; ##	54.7 ± 10.2***	135.9 ± 15.1***; #
$LaVO_4:Eu^{3+}$ (50 mg/L)	6.9 ± 2.8	6.5 ± 1.8	7.7 ± 2.8
$LaVO_4:Eu^{3+}$ (50 mg/L) + tBOOH	172.6 ± 17.4***; ##	59.3 ± 12.1***	188.4 ± 15.9***; ##

Note: ANOVA and Tukey's tests, mean ± SEM (n = 3). Note: * ($p < 0.05$); ** ($p < 0.01$); *** ($p < 0.001$) compared with the control samples; # ($p < 0.05$); ## ($p < 0.01$); ### ($p < 0.001$) compared with the H_2O_2- or tBOOH-treated samples. Rfu – relative fluorescence units.
